# Strong Families Start at Home/Familias Fuertes Comienzan en Casa—Improving Child Diet Quality and Parental Feeding Practices: Protocol for a Randomized Controlled Trial

**DOI:** 10.2196/73923

**Published:** 2025-09-10

**Authors:** Alison Tovar, Kelly Lynn Bouchard, Amy M Moore, Michelle Perry, Ivone Lurssen, Leonardo Arriola Carnicelli, Alexia Sanchez Contreras, Patricia Risica, Tayla von Ash, Jennifer S Savage, Shira Dunsiger, Kim Gans

**Affiliations:** 1 Department of Behavioral and Social Sciences School of Public Health Brown University Providence, RI United States; 2 Department of Nutritional Sciences College of Health and Human Development Pennsylvania State University University Park, PA United States; 3 Institute for Collaboration on Health, Intervention, and Policy University of Connecticut Storrs, CT United States

**Keywords:** Hispanic or Latino, child, diet quality, feeding practices, feeding behavior, randomized controlled trial, motivational interviewing

## Abstract

**Background:**

Children in the United States have poor diet quality, increasing their risk for chronic disease burden later in life. Caregivers’ feeding behaviors are a critical factor in shaping lifelong dietary habits. The Strong Families Start at Home/Familias Fuertes Comienzan en Casa (SFSH) was a 6-month, home-based, pilot randomized-controlled feasibility trial that aimed to improve the diet quality of 2-5-year-old children and promote positive parental feeding practices among a predominantly Hispanic/Latine sample. The pilot saw significant improvements in children’s Healthy Eating Index-2015 total and whole fruit scores, as well as multiple food parenting practices, and it was received well by participants.

**Objective:**

Building on the success of the pilot study, this protocol paper describes the modifications, study design, and procedures for a fully powered randomized controlled trial.

**Methods:**

Caregiver-child dyads are randomized to a “healthy eating” intervention group or a “reading readiness” attention control group. In the intervention group, a trained community health worker conducts monthly home visits or phone calls for 6 months that focus on age-appropriate nutrition recommendations and food parenting practices. There are three home visits that include tailored nutrition education materials that address their child’s appetitive traits and eating habits, an interactive cooking activity, and a review of a family meal video with feedback. Community health workers use motivational interviewing and goal setting, which are key components of the program. The control group is similarly structured, with content focusing on reading and language development. Caregivers complete in-person and over-the-phone baseline and 6-month follow-up measurements to capture diet quality (primary outcomes: Healthy Eating Index-2015 scores via two 24-h dietary recalls and dermal carotenoids) and selected parental feeding practices and availability of healthy foods in the home (secondary outcomes).

**Results:**

This protocol was approved by the Brown University institutional review board (protocol number 2022003389). As of March 2025, a total of 81 participants were randomized. Of these, 29 participants completed the study, and 8 participants withdrew. Recruitment will continue until 257 participants have been randomized. Data analysis is expected to conclude in 2028.

**Conclusions:**

Findings will determine the efficacy of the intervention to improve child diet quality and parental feeding practices, which will ultimately inform future effectiveness and the real-world of home-based food parenting programs.

**Trial Registration:**

ClinicalTrials.gov NCT06099288; https://clinicaltrials.gov/study/NCT06099288

**International Registered Report Identifier (IRRID):**

DERR1-10.2196/73923

## Introduction

Dietary behaviors across the lifespan affect the risk of developing cardiovascular disease, type II diabetes, certain cancers, and Alzheimer disease, among other prominent chronic diseases [1-3]. Of these conditions, Hispanic/Latine adults who reside in the United States are disproportionately impacted by type II diabetes, hypertension, and end-stage renal disease compared to White adults, but they have poorer access to health care services [4,5]. Early childhood is a critical period for establishing a foundation for healthy dietary patterns that promote long-term quality of life and well-being [6-13]. However, the average diet quality among children in the United States is suboptimal [14,15], with notable disparities across racial and ethnic groups, as well as varying socioeconomic statuses [16,17]. Young Hispanic/Latine children in particular consume fewer fruits and vegetables and more frequently drink sugar-sweetened beverages than White children [18]. While broader policies and environmental factors influence children’s dietary patterns and behaviors, the home—where children spend much of their time—offers a unique opportunity for targeted, culturally relevant interventions to promote healthy eating habits.

Food parenting practices play a significant role in shaping children’s dietary intake and long-term eating behaviors. Current recommendations that promote the use of structured and autonomy supportive parenting practices, such as role modeling healthy eating, providing consistent access to nutritious food, establishing mealtime routines, and autonomy support, are strongly associated with improved dietary outcomes and child appetite regulation [19-22]. For example, a systematic review showed that parental role modeling positively influences children’s fruit and vegetable consumption, as children learn by observing their caregivers’ eating behaviors [23]. Specific to food availability, increased availability of fruits and vegetables in the home environment leads to higher consumption of these foods by children [24]. Finally, studies emphasize that autonomy-supportive practices, which include encouraging independence and healthy eating through strategies like praise, nutrition education, and reasoning, encourage children to try new foods, willingness to adopt healthy eating habits, and variety in dietary intake [25].

Conversely, the use of coercive controlling practices that dominate a child’s behavior is discouraged due to their potential to negatively influence children’s relationship with food. Practices such as using food as a reward or punishment, imposing food restriction, or engaging in emotional feeding have been linked to maladaptive eating behaviors, including overeating or aversions to specific foods [26,27]. For example, food restriction may be counterproductive, resulting in increased desire for and consumption of the restricted foods when children have access to them, potentially contributing to overweight [28]. Similarly, using food to soothe distress or manage child behavior can lead to an unhealthy association between emotions and eating [29]. These findings suggest the importance of adopting evidence-based, supportive food parenting practices to create a home food environment that promotes healthy dietary patterns and eating behaviors.

Food parenting interventions for preschool-aged children have focused on improving dietary behaviors such as increasing fruit and vegetable consumption and reducing sugary beverages and energy-dense snacks. For example, Healthy Habits, Happy Homes promoted structured meal routines, parental modeling, and reduced screen time, resulting in modest improvements in children’s dietary behaviors, including more frequent family meals and decreased sugary drink intake [30]. Similarly, the NOURISH trial demonstrated responsive feeding practices, taught parents to reduce coercive feeding strategies, and encouraged positive behaviors that promote better self-regulation and openness to trying new foods. While these approaches improved feeding practices, changes in children’s dietary intake were modest and inconsistent over time [31].

An updated 2024 Cochrane systematic review by Hodder et al [32] reported that child-feeding interventions that incorporate repeated food exposure led to small but positive increases in vegetable consumption. Previous iterations of this review also found that multicomponent interventions involving parents and children, including taste exposure, role modeling, and skill-building, resulted in small but measurable improvements in fruit and vegetable consumption [33]. Despite these promising findings, gaps persist. Many programs lack tailoring to children’s individual needs and fail to be culturally appropriate. These shortcomings highlight the need for co-designed, community-informed approaches that are adaptable, culturally sensitive, and capable of addressing the unique dietary and environmental contexts of diverse populations.

Strong Families Start at Home/Familias Fuertes Comienzan en Casa (SFSH; ClinicalTrials.gov NCT03923491) was a home-based pilot intervention specifically designed for low-income, predominantly Hispanic/Latine parents of 2- to 5-year-old children [34]. Its primary components included identifying and enforcing positive parental feeding practices using motivational interviewing (MI), overcoming feeding challenges by acknowledging children’s unique appetitive traits, and reviewing food shopping and preparation strategies. SFSH responded to the need for culturally relevant, tailored interventions that reach caregivers at home, where many behavioral decisions are made. This paper describes the current expansion of the SFSH intervention, modifications to its design, and implementation (ClinicalTrials.gov NCT06099288).

## Methods

### Aims

The primary aim of the intervention is to examine changes in child diet quality between the baseline and 6-month follow-up visits. Secondary aims include assessing for improvements in food parenting practices and the availability of healthy foods in the home. Additional exploratory aims include exploring the relationships between outcome measures and potential mediators and moderators of these effects. The trial has been registered at ClinicalTrials.gov ID NCT06099288.

### Recruitment, Eligibility, and Data Collection

This study targets primary caregivers of 2-5-year-old children who identify as Hispanic/Latine. Additional eligibility criteria include being at least 18 years of age, being able to speak English or Spanish, having a smartphone, and being willing to receive SMS text messages and record a family meal or reading activity. Participants are excluded from the study if their child has a diagnosed eating or feeding disorder or if they participated in the pilot. Participants with multiple children in the 2- to 5-year age range may only enroll with one child. We aim to recruit 257 participants to achieve adequate power for the proposed data analysis (see sample size calculations).

Different aspects of community engagement are critical to the recruitment success of this study. The first is working with our two community partners, and the second is spending time in the community. Participants are mainly recruited by study personnel at food pantries and other community organizations in Rhode Island who administer an eligibility screener and collect contact information. We also work closely with our community partners to share information and distribute fliers, which have a quick response code that directs to the eligibility screener when scanned. Interested participants answer a series of eligibility questions. If they do not meet the requirements, they receive an immediate notification informing them of their ineligibility. If they qualify, they are notified that a research assistant (RA) will follow up with them within the next few weeks. We also have a study website where participants can learn more about the study and fill out the eligibility screener [35].

Baseline data collection occurs in three phases. First, a bilingual RA calls the individual to obtain their oral consent to participate in the study with their child. The RA then administers some of the baseline questionnaire during the same call, as time allows. Both participant consent and questionnaire data are documented in the Qualtrics survey platform. Next, the RA goes to the participant’s home to finish the questionnaire; obtain the child’s height, weight, and dermal carotenoid score; and conduct a 24-hour dietary recall. Finally, the participant completes a second dietary recall over the phone within 2 weeks of the baseline visit.

Participants are randomized after completion of the second dietary recall. A randomization scheme was created by the study’s statistician based on a permuted block randomization procedure with small random-sized blocks. Group allocations were put into opaque, numbered envelopes by a team member who is not involved in data collection. Once a participant has been randomized, they are assigned to a community health worker (CHW) who schedules all visits and phone calls until follow-up.

The project coordinator is unblinded to the group assignments of all participants, and the CHWs are aware of the group assignments for the participants to whom they deliver the intervention. However, the personnel responsible for collecting data are blinded to the participants’ group assignments. While participants are aware of the content of the material they are discussing, they are informed that the intervention topics are “healthy eating” and “food parenting,” while “reading readiness” is the control topic.

### Ethical Considerations

This protocol was approved by the Brown University institutional review board (protocol number 2022003389). Contact information is collected in the eligibility screener. This is stored in Ripple, which is a HIPAA (Health Insurance Portability and Accountability Act)-compliant platform. An RA reviews an informed consent document with the participant over the phone. The document specifies that participation is voluntary and that participants may withdraw from the study at any time without penalty. Verbal informed consent to participate is obtained over the phone in the participant’s preferred language (English or Spanish) and is documented by the RA in Qualtrics. Participants are provided with a physical copy of the informed consent document at the baseline visit. The verbal informed consent form in Qualtrics contains the participant’s name, the participant’s child’s name, and an ID number. No other identifying information is collected from our data collection instruments. The data are instead associated with the ID number and are stored in password-protected platforms and storage services that require a Brown University account to access. All data will remain anonymized when analyzed and published. Participants are reimbursed through a prepaid debit card that study personnel add funds to at predetermined study milestones. A total of US $40 is added to the card for completing the baseline visit, and an additional US $20 is added after the second dietary recall. Participants then receive US $40 and US $100 for completing the third home visit and all follow-up data collection, respectively.

### Overview of Design

**Figure 1:**
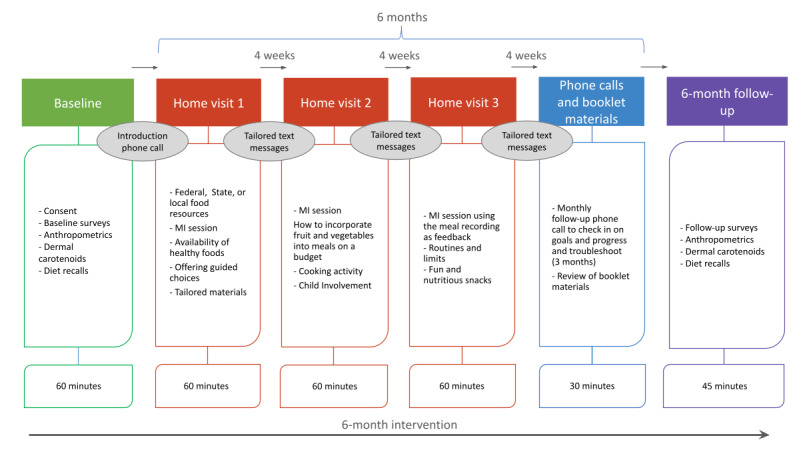
SFSH study design. MI: motivational interviewing; SFSH: Strong Families Start at Home/Familias Fuertes Comienzan en Casa.

SFSH’s pilot design has been described previously [34,36]. The program and performance objectives we identified through intervention mapping for the pilot, as well as the overall structure of the intervention, remain unchanged (Figure 1) [37]. Briefly, SFSH is a 6-month randomized controlled trial. Participants engage in three monthly 60-minute in-home sessions followed by three monthly 30-minute phone calls. These sessions are led by a bilingual (Spanish and English) CHW. Each CHW receives onboarding training in basic principles of pediatric nutrition and parental feeding practices from the project coordinator, who is a registered dietitian. Ongoing booster training is provided every 6 to 8 weeks. The CHWs also undergo MI certification training led by a member of the study team who has extensive experience in MI (AMM). The CHWs meet with AMM monthly for ongoing MI support.

Participants receive a booklet containing study content, tailored information about eating behaviors, information about nutrition, and biweekly SMS text messages that reinforce topics covered during the in-home and phone sessions. All calls and SMS text messages are made through a single shared phone number that is hosted on the Avochato platform.

### Theory

**Figure 2:**
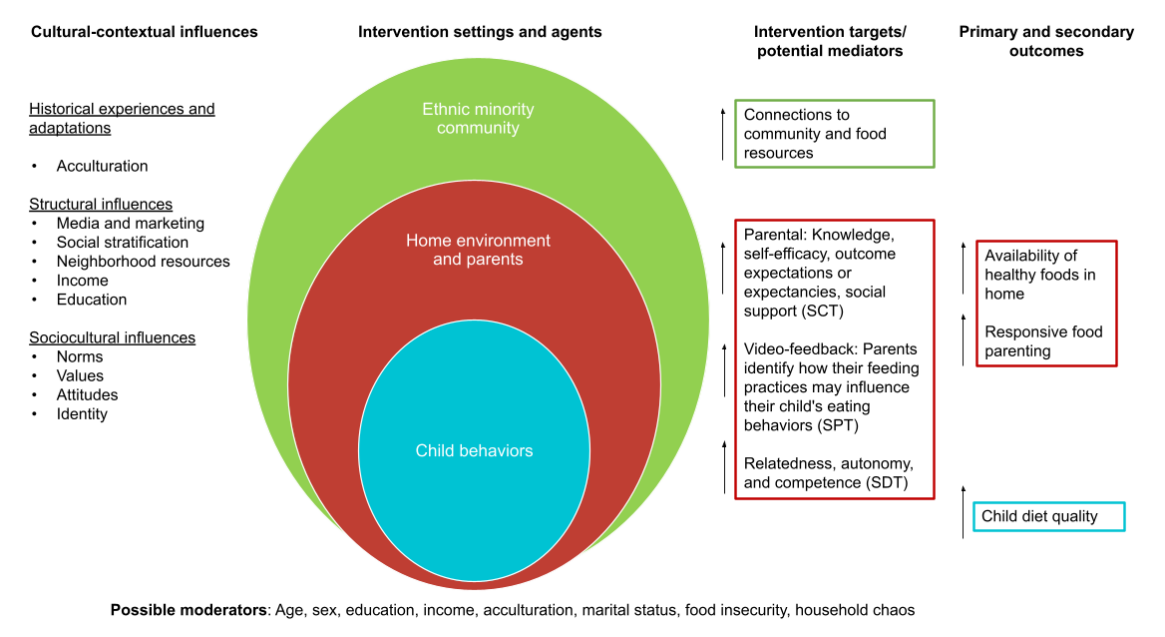
Conceptual and logic model guiding intervention. SCT: social cognitive theory; SDT: self-determination theory; SPT: self-perception theory.

Consistent with our pilot, three behavioral theories guided the development of the SFSH intervention (Figure 2). First, social cognitive theory posits that behavior is shaped by bidirectional relationships between intrapersonal, social, and environmental factors [38,39]. Parental self-efficacy, behavioral capability, and outcome expectations and expectancies are elements of social cognitive theory that SFSH targets to evoke change in child diet quality and parental feeding practices. Second, self-determination theory describes autonomy, competence, and relatedness to others as drivers of internal motivation, which is considered to be more constructive than external motivators [40]. Our intervention leverages CHW-led MI to foster these components, thereby facilitating desired behavior change and maintenance [41]. Finally, the video feedback activity during the third home visit is informed by self-perception theory, which states that individuals gain insight into their attitudes and beliefs when they observe their own behaviors and the context in which they were performed [42]. In SFSH, participants are able to reflect on their feeding practices after reviewing a self-recorded video of a family meal.

### Participant Considerations

Several measures were taken to promote the cultural relevance of SFSH for Hispanic/Latine caregivers. First, insights from the pilot study’s focus groups that were majority Hispanic/Latine (28/33, 85%) were incorporated into the development of this study [36]. Second, feedback obtained from the pilot’s follow-up survey and postintervention semistructured interviews was also considered when updating materials and protocols [34]. Third, we established a parent leader board consisting of six Hispanic/Latine parents in the community to review materials for this study, in addition to a leadership community advisory board made up of our community-based organization leadership, SNAP-Ed, and WIC representation. Finally, all study personnel who speak with participants, including CHWs and RAs, identify as Hispanic/Latine and are fluent in Spanish.

**Figure 3:**
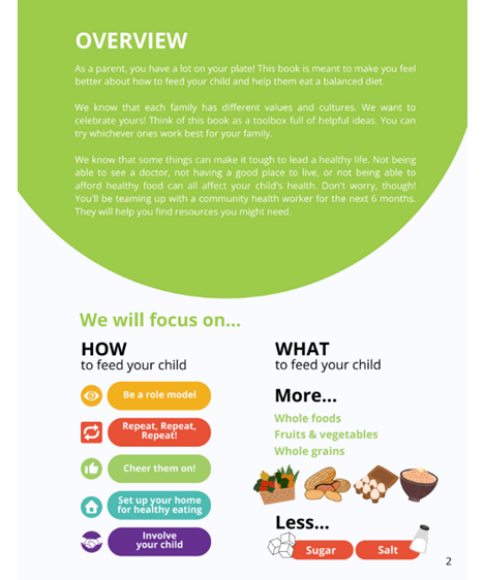
Sample 1: page from the Strong Families Start at Home booklet.

**Figure 4:**
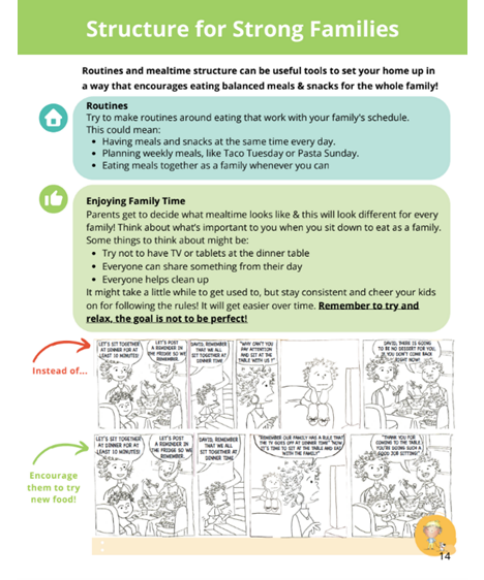
Sample 2: page from the Strong Families Start at Home booklet.

Literacy level is not part of our eligibility or exclusion criteria. All materials have been written at a 6th-grade reading level to make them more accessible to low-literacy participants. We have also incorporated visuals and comics into our materials to support retention and understanding of key concepts (Figures 3 and 4). If participants share that they are unable to read English or Spanish during screening, consenting, or the baseline data collection process, an RA verifies that someone in the home can assist with reading and writing as needed.

### Intervention Components

Prior to the first home visit is a 15-minute introduction phone call. This call was added to the protocol to allow the CHWs to build rapport with the participant before meeting in person. During this call, the CHW also administers a 7-item social determinants of health (SDOH) screener that we adapted from the Accountable Health Communities Health-Related Social Needs Screening Tool (Table 1) [43]. CHWs then connect participants who request help with identified needs to relevant community resources.

**Table 1:** Social determinants of health screener.

	Questions					Response options
Health care						
			Think about your health care needs. Do you have problems with any of the following?		1. Health insurance 2. Accessing medications 3. Quality health care 4. None of the above	
Housing						
			What is your housing situation today?		1. I do not have housing 2. I have housing today, but I am worried about losing housing in the future 3. I have housing	
		Think about the place you live. Do you have problems with any of the following?			1. Bug infestation 2. Mold 3. Lead paint or pipes 4. Inadequate heat 5. Oven or stove not working 6. No or not working smoke detectors 7. None of the above	
Food						
			Within the past 12 months, you worried that your food would run out before you got money to buy more.		1. Often true 2. Sometimes true 3. Never true	
				Within the past 12 months, the food you bought just did not last, and you did not have the money to get more.		1. Often true 2. Sometimes true 3. Never true
Transportation						
				In the past 12 months, has a lack of transportation kept you from medical appointments, meetings, work, or getting things needed for daily living?		1. Yes, it has kept me from medical appointments or getting medications 2. Yes, it has kept me from nonmedical meetings, appointments, work, or getting things that I need 3. No
Utilities						
				In the past 12 months, has the electric, gas, oil, or water company threatened to shut off services in your home?		1. Yes 2. No 3. Already shut off
Assistance						
				Would you like help with any of these needs?		1. Yes 2. No

Over 6 months, three 60-minute home visits are completed with participants, followed by three 30-minute phone calls. The participant is given a booklet at the first home visit, which contains sections to be reviewed at each visit and phone call. The content focuses on five parenting strategies to promote healthful eating practices: (1) role modeling, (2) repeated exposure, (3) encouraging language, (4) structuring the home environment, and (5) child involvement. The booklets also contain comic strips featuring these strategies; Specific, Measurable, Achievable, Relevant, and Time-Bound goal templates; general nutrition guidelines for young children; and meal planning tips (Figures 3 and 4). One or two food parenting topics are reviewed at each visit and phone call, then the CHW uses MI to help the participant set goals around the content they covered. These goals are viewed at subsequent visits and calls.

Each home visit features a supplementary activity tailored to the individual needs of their child. At the first home visit, participants may be given handouts that address one of their child’s appetitive traits or dietary habits based on their responses to the Children’s Behavioral Eating Questionnaire and questions adapted from the National Survey of Children’s Health, respectively [44-46]. Both measures are administered during baseline data collection and are scored prior to the first home visit. The Children’s Behavioral Eating Questionnaire is a validated caregiver-reported tool consisting of eight appetitive trait subscales. As in the pilot, participants whose children meet the criteria for food fussiness, satiety responsiveness, or food responsiveness are provided with a tailored handout that lists strategies to encourage healthy eating habits [34,36,47]. If the child exhibits more than one trait, information is prioritized in the aforementioned order. In addition to addressing the child’s eating behaviors, the family receives an additional handout if the child has a high intake of sugar-sweetened beverages (≥4-6 times in the past week), energy-dense snack foods (≥4-6 times in the past week), or low fruit and vegetable intake (≤1 time per day). These behaviors were selected because they are among the most prevalent in children nationally and are strongly associated with adverse health outcomes [48].

The second home visit is largely unchanged from the pilot [36]. Briefly, a culinary professional from the community accompanies the CHW to lead an interactive cooking demonstration during the second home visit to provide guidance on basic culinary skills and age-appropriate ways to involve children in food preparation. Participants are also given a cookbook with culturally relevant recipes and child cooking utensils.

The third home visit features a video feedback activity. Participants are asked to record and send a 3- to 5-minute video of a typical family meal to study personnel at least a week prior to the next visit. An RA then codes the video for structured or autonomy supportive and nonstructured or nonautonomy supportive parental feeding practices. The coding tool was developed specifically for the pilot based on the constructs defined by Vaughn et al [25]. Six experts in food parenting reviewed this tool and provided feedback, and it demonstrated good interrater and excellent test-retest reliability [49]. Ultimately, one structured or autonomy supportive and one nonstructured or nonautonomy supportive practice is clipped and played back to the participant. Using MI, the CHW asks participants about their observations and impressions about the clips during the home visit to facilitate open-ended discussion. The RA also prepares a feedback sheet that explains why the clips were selected, how they may impact children’s eating habits, and tips on how to implement more positive practices, which the CHW reviews with the participants after the participants share their initial thoughts. Example video clips and feedback sheets are prepared for participants who do not send their own.

Following the home visits are three monthly 30-minute phone calls. The calls are structured similarly to the home visits: the CHW assesses progress toward the previously set goal, discusses content from the study booklet, and helps participants set a new goal. Much of the content reviewed during the phone calls reinforces lessons from the home visits. Participants who notify their CHW that they have lost their booklets are mailed copies of the appropriate pages prior to their next call. The CHW also readministers the SDOH screener at the end of the first phone call to reevaluate and address participant needs.

Participants receive automated SMS text messages twice weekly via the messaging software Avochato for the duration of the intervention, starting after the first home visit. These contain links to recipes, advice on positive parental feeding, and general motivational phrases.

The control arm of this study is an attention-control program that promotes literacy and school readiness, which was derived from the Greater Washington Educational Television Association’s Reading Rockets curriculum [50]. Participants in this group have the same home visit and phone call schedule as the intervention. However, they do not receive tailored information at the first home visit. In place of the in-home cooking activity, the CHW leads a poem activity with the participant and their child and provides three storybooks as incentives. Rather than reviewing a family meal during the third home visit, participants instead have a video feedback session where they review clips of themselves reading to their child. They also sent SMS text messages twice weekly, featuring language that is consistent with the control protocol.

### Modifications From Pilot Study

While our pilot study results were promising, there were some modifications that were made to this study to further improve recruitment, retention, and better meet the needs of the community. We provide a brief overview of these changes here. First, the CHWs felt that it was important to have time to get to know the participants prior to going into their homes, so we added a 15-minute phone call prior to the first home visit. Second, many families from the pilot study faced challenges related to SDOH. Without having some of their basic needs met, it is hard to expect that they would prioritize their children’s feeding. Thus, we added an SDOH screener to the first phone call so that CHWs could help provide referrals and resources to families. We felt it was important to ground our work within the community energy balance framework to address the cultural and contextual influences predisposing Latine families to unhealthy diets. Through MI, the CHW builds rapport and identifies some of the structural and sociocultural influences that impact their families’ eating (Figure 2). Third, we wanted to ensure that we were listening to parent voices and felt that just having a community advisory board with leadership from community-based organizations (which we continued) was not sufficient. As a result, we created a parent leader board, in addition to a leadership community advisory board, made up of six Hispanic/Latine parents to provide feedback on study materials, advise on the cultural relevance, and effective dissemination and retention strategies on an ongoing basis. Fourth, we decided to add additional tailored information to the intervention based on parents’ baseline reports of their child’s dietary intake. Fifth, we added a biomarker to measure a child’s dietary intake (dermal carotenoids measured by the Veggie Meter) to supplement other dietary measures. Finally, we decided to work closely through contracting with two community-based organizations that work with families in Rhode Island. They participate in our leader community advisory board, and we have ongoing meetings to discuss recruitment in the community.

### Justification of Design

SFSH’s principal components bring together three motivational theories. MI is especially critical—it has been incorporated into every monthly session due to its effectiveness in promoting sustainable behavior change across a range of settings and contexts [51]. Therefore, personnel must undergo MI certification training to ensure that it is being used accurately. These features and training requirements may make it challenging for low-resource settings to adopt this program. Some adaptations can be made to accommodate limited materials without compromising fundamental aspects if needed. For example, booklet and handout content can be sent to participants’ smartphones in place of providing physical copies to eliminate printing costs. Home visits can also be conducted remotely.

### Measures and Outcomes

#### Primary Outcome

The primary outcome of this trial is child diet quality using the Healthy Eating Index-2015 (HEI) scores that are calculated from multiple-pass 24-hour dietary recalls, a highly valid method for capturing dietary intake [52]. To improve the accuracy of reporting, participants are given a bilingual food amounts booklet at the baseline visit to help them visualize quantities of the foods and beverages they consumed [53]. Participants are also asked to reference this guide during the second recall if possible. Recalls are scheduled to capture one weekday and one weekend day to obtain a more accurate representation of the child’s typical intake. If the child has spent a portion of the recall period away from the participant and the participant is unable to provide enough information on the types and quantities of foods eaten during those times, participants are asked to reach out to who the child was with to gather this information if possible. In the case of preschool and childcare facilities, participants are asked to obtain the menu that was used for the day that is being recalled. If the menu is not available, a sample menu from the Child and Adult Care Food Program is used. All reported items are entered into the 2022 Minnesota Nutrient Database for Nutrition Research (NDSR) [54]. Nutrient data provided by NDSR from both days are then averaged and used to generate HEI scores.

The HEI is a scale that reflects adherence to the Dietary Guidelines for Americans [55]. It is comprised of 13 components: total fruits, whole fruits, total vegetables, greens and beans, whole grains, dairy, total protein foods, seafood and plant proteins, fatty acids, refined grains, sodium, added sugars, and fatty acids. Points from each component are summed to a total score that can range from 0 to 100, with higher scores indicating better diet quality. HEI scores will be calculated directly by NDSR. All dietary recalls will be assessed for quality assurance by study personnel prior to data analysis.

We are also using dermal carotenoids as a proxy measure of diet quality. Carotenoids are a classification of red, orange, and yellow naturally occurring pigments produced by plants, bacteria, and fungi. These compounds accumulate in the skin after consumption [56]. Hence, they may be used as a biomarker to approximate fruit and vegetable intake. The Veggie Meter (Longevity Link) noninvasively measures dermal carotenoids via reflection spectroscopy [57]. This device has been validated to detect changes over time among racially and ethnically diverse samples [58,59], and other studies have used it to assess dermal carotenoid levels among preschool-aged children [60,61]. Participants are asked to guide their child’s finger onto the lens of the Veggie Meter. The average of three 10-second measurements with two 10-second washouts between is then recorded. Higher scores indicate higher dermal carotenoid levels, with the maximum score being 800.

#### Secondary Outcomes

All the measures that assessed parental feeding practices in the pilot will be used here. The 13 subscales of the Food Parenting Inventory (child involvement in food preparation, responsiveness to child’s fullness cues, encourage exploration of new foods, repeated presentation of new foods, family meals, regular timing of meals and snacks, encourage trying new foods, inconsistent mealtimes, indifferent feeding, pressure to eat, restriction, food as a reward, and monitoring) and the Healthy Eating Guidance subscale from the Comprehensive Feeding Practices Questionnaire have both demonstrated reliability and validity among Latine caregivers specifically [62-64]. The former was developed to consolidate assessments of multiple parental feeding practices into one tool, and the latter was developed to capture previously less explored feeding practices such as restrictive feeding. The Parent Socioemotional Context of Feeding Questionnaire was developed based on the constructs of self-determination theory to evaluate socioemotional aspects of the home feeding environment and has been validated for use with caregivers of 4- to 8-year-old children [65]. Items from all three questionnaires are measured on 5-point Likert scales ranging from 1=Never to 5=Always, 1=Disagree to 5=Agree, or 1=Extremely untrue to 5=Extremely true, as applicable. Higher scores indicate greater compliance with the feeding practice or construct that corresponds to each question. Additionally, a single-question family meal frequency measure taken from a systematic review of family meal assessments is included [66].

A home food inventory is used to compare pre- and postintervention availability of healthy and unhealthy foods in the home [67]. Our modified version is a 20-item “Yes/No” checklist inquiring about fruit, vegetables, chips, sugary snacks or desserts, candy, beverages, whole grains, and beans and legumes.

#### Potential Moderators and Mediators

Data on potential moderators is being collected at baseline and include caregiver age, gender, race, marital status, income, birth country, number of years living in the United States, household composition, acculturation [68], food and nutrition insecurity [69,70], and participation in federal nutrition programs, as well as child age, gender, and time spent in childcare. Needs identified by the SDOH screener that is administered at the introduction and month 4 phone calls will also be considered [43].

We will explore several potential mediators that the SFSH pilot was not sufficiently powered to analyze. Parent diet quality is measured by the 15-item Rapid Prime Diet Quality Score Screener, as well as parent dermal carotenoid levels [71]. Nine questions were selected from a food literacy behavior checklist to evaluate engagement in meal preparation and healthy mealtime practices [72]. We are assessing selected constructs of the theoretical frameworks that informed this intervention’s design: perceived barriers to healthy eating using the National Cancer Institute’s Food Attitudes and Behaviors Survey [73], perceived familial support using the short form Family Health Scale [74], and relatedness and competence with a tool we adapted from the Intrinsic Motivation Inventory [75,76].

A language checklist was developed by the study team to capture potential changes in reading or communication skills in the control group.

### Process Evaluation

To capture intervention dose, we are reviewing the total number of completed home visits and phone calls, SMS text messages, and materials that were read as reported by participants, and referrals to social services. Additionally, following each session, CHWs submit a brief process measure that assesses participant responsiveness to the intervention. Participants are also asked to complete a satisfaction survey upon study completion.

Implementation fidelity is being monitored by trained study team members across three main domains (adherence, quality of delivery, and participant responsiveness). A subsample of audio recordings from the intervention and control group sessions is reviewed for adherence (eg, was the intervention delivered as intended) and quality of delivery (eg, manner in which the intervention was delivered) across the study. Shortly after study launch, a small sample (n=7) of session recordings was reviewed for all CHWs to monitor adherence. Recordings are scored using a study team–developed implementation checklist that includes binary response options (yes/no) for the main intervention components. To assess initial fidelity to the protocol, five recordings of each intervention home visit and phone call, and two recordings for each control home visit and phone call are reviewed for each CHW. If CHWs score below 80%, booster trainings are delivered. Monitoring includes review of five randomly selected recordings for each CHW every 3 months. In addition, shortly after the study launch, a recording was reviewed for each CHW to monitor adherence and quality of delivery for MI. A random 20-minute segment of each recording was reviewed and coded using the Motivational Interviewing Treatment Integrity Coding Manual (MITI 4.2.1) to assess beginning proficiency [77]. The first five intervention recordings and the first two control recordings completed by each CHW for each home visit and phone call will be coded for MI adherence and quality. A random sample of five recordings for each CHW will then be coded every 3 months for the duration of the study. CHWs receive personalized feedback, and MI meetings are conducted to build skills and prevent drift.

### Sample Size Calculations

We have conducted power calculations to ensure that we have an adequate sample size such that between-group differences can be detected in the primary outcome of diet quality measured by 24-hour dietary recalls and the HEI-2015. Power was calculated using a combination of G*Power (Heinrich Heine University Düsseldorf) and MPlus Monte Carlo estimation informed by the pilot study, which showed effects favoring intervention on median changes in HEI from baseline to end of treatment (0.69 vs –6.48), corresponding to a medium effect size (*f*^2^*=*0.14). We have chosen to power on medians (instead of means) due to the high variability around the mean (which is common in smaller samples). We anticipate seeing a greater average change in HEI than what we saw in our R34, given the strengthening of our intervention and that the pilot was conducted at the beginning of the COVID-19 pandemic. We also expect significantly less variability in a larger sample. As our planned analysis is to consider differences in mean HEI between groups over time, we have also conducted simulations to ensure we would be adequately powered given medium-sized effects in mean HEI between groups over time (*f*^2^=0.14). We expect that our proposed intervention will improve total HEI by 5 units [78]. We selected five HEI units based on the following rationale: (1) five HEI units is clinically meaningful as it predicts a 4%-6% decrease in overall mortality and a 15% decrease in the prevalence of obesity [79,80]; (2) five HEI units is statistically meaningful as it is approximately 0.5 of the SD of HEI when measured in large, representative samples; and (3) five HEI units is a reasonable expectation for a moderately intensive, intervention, with previous studies reporting increases from 3.6 to 7.8 [81].

For this study, with 180 participants at follow-up, we will have >85% power to detect differences in primary outcome variables (HEI) between groups (I vs C), using a 2-tailed significance level α=0.05 and effect sizes consistent with our prior work. To confirm adequate power, simulation studies were run using a series of mixed effects Monte Carlo simulations with 1000 replications and three seeds to confirm model stability. Models assumed modest effects of covariates and a range of effect sizes for both the main condition and covariates. Findings supported >85% power to detect small to medium effects (*f*^2^<0.13). Although analysis will be based on the intent-to-treat sample, we plan to enroll 257 participants to ensure a final sample of 180 participants. In our pilot study, of those recruited, we had a 75% enrollment rate.

### Statistical Analysis

Between-group differences in baseline demographic characteristics will be compared using a combination of parametric and nonparametric tests as appropriate. Potential confounders identified through this process will be included in subsequent models. Primary and secondary outcomes will be examined using a series of linear mixed effects models that include group assignment, baseline values of the outcome, intervention dose received, and confounders identified a priori. We will use a similar analytic strategy to examine potential moderators. In this case, models will include the main effects of the moderator, group, and the moderator by group. Mediators will be analyzed using multiple mediator models implemented with a product of coefficients approach with bootstrapped standard errors. Missing data will be approached on the intent-to-treat principle and compared using both likelihood-based approaches to estimation and inverse probability weighting and pattern mixture models as appropriate.

## Results

The notice of award from the National Institutes of Health was received in March 2023. We revised protocols, updated study materials, hired and trained study personnel, prepared data management platforms, and engaged in outreach with community partners between March 2023 and January 2024. Recruitment began in February 2024. A total of 81 participants were randomized as of March 2025. Of these, 44 participants are completing the program (intervention: n=22 intervention; control: n=22), 29 participants have completed follow-up data collection (intervention: n=13; control: n=16), and 8 participants have withdrawn (intervention: n=5; control: n=3).

Data analysis is expected to conclude in 2028. Once the main results are analyzed, we plan to disseminate our findings back to the research participants through a one-page summary written in lay terms, as well as at community events, particularly with our community partners and our community advisory boards. We will also publish our results in the peer-reviewed literature and share results with relevant stakeholders such as SNAP-Ed, WIC, or home visiting.

## Discussion

### Principal Findings

Novel interventions are required to address disparities in diet quality that are prevalent among populations commonly affected by social inequities. The SFSH pilot was a feasible and acceptable home-based nutrition intervention among a racially and ethnically diverse sample of caregivers of preschool-aged children. Furthermore, we observed improvements in multiple HEI subscale scores and parental feeding practices [34]. This study expands on the pilot and features numerous modifications to improve these outcomes among Hispanic/Latine families.

There are several challenges we may encounter throughout the study. First, it may be difficult to recruit and retain participants in our 6-month program. However, despite the COVID-19 pandemic necessitating changes in study procedures in the pilot, we were successful in recruiting our target number of participants. We continue to rely on the community partnerships that aided us in our prior recruitment efforts; therefore, we do not anticipate greater difficulty in this trial. To improve retention, we have increased our total incentive amount to US $200, which will be distributed in segments at prespecified study milestones. Additionally, all members of the study team can communicate with participants through Avochato, enabling us to more easily send visit reminders and respond to their inquiries.

Another potential challenge is participants not recording videos for the third home visit. In the pilot, 62% (24/39, intervention group) of participants submitted a video for the tailored feedback activity. For this intervention, we created instructional videos depicting how to record and send the feeding videos. A text reminder is sent 2 weeks prior to the third home visit that reiterates these instructions. Participants who send their videos are entered into a raffle to win an extra incentive. If participants still fail to send a video, we created sample videos and feedback sheets that they can review.

### Conclusions

This expansion on the SFSH pilot enhances its potential to address the significant public health concern of nutrition-related chronic disease burden among the Hispanic/Latine population. Our novel theory and home-based approach are intended to empower caregivers to make lasting changes to their feeding practices and support healthy eating in their homes. Integrating tailored information further helps families navigate unique nutritional and eating-related barriers. SFSH’s promising start suggests that it could be incorporated into community programs.

## Abbreviations

CHW: community health worker
HEI: Healthy Eating Index
HIPAA: Health Insurance Portability and Accountability Act
MI: motivational interviewing
MITI: Motivational Interviewing Treatment Integrity Coding Manual
NDSR: Minnesota Nutrient Database for Nutrition Research
RA: research assistant
SDOH: social determinants of health
SFSH: Strong Families Start at Home/Familias Fuertes Comienzan en Casa
